# Identification of *Burkholderia pseudomallei* Near-Neighbor Species in the Northern Territory of Australia

**DOI:** 10.1371/journal.pntd.0003892

**Published:** 2015-06-29

**Authors:** Jennifer L. Ginther, Mark Mayo, Stephanie D. Warrington, Mirjam Kaestli, Travis Mullins, David M. Wagner, Bart J. Currie, Apichai Tuanyok, Paul Keim

**Affiliations:** 1 Center for Microbial Genetics and Genomics, Northern Arizona University, Flagstaff, Arizona, United States of America; 2 Menzies School of Health Research, Casuarina, Northern Territory, Australia; 3 Department of Infectious Diseases and Pathology, University of Florida, Gainesville, Florida, United States of America; 4 Pathogen Genomics Division, Translational Genomics Research Institute, Flagstaff, Arizona, United States of America; Beijing Institute of Microbiology and Epidemiology, CHINA

## Abstract

Identification and characterization of near-neighbor species are critical to the development of robust molecular diagnostic tools for biothreat agents. One such agent, *Burkholderia pseudomallei*, a soil bacterium and the causative agent of melioidosis, is lacking in this area because of its genomic diversity and widespread geographic distribution. The *Burkholderia* genus contains over 60 species and occupies a large range of environments including soil, plants, rhizospheres, water, animals and humans. The identification of novel species in new locations necessitates the need to identify the true global distribution of *Burkholderia* species, especially the members that are closely related to *B*. *pseudomallei*. In our current study, we used the *Burkholderia*-specific *recA* sequencing assay to analyze environmental samples from the Darwin region in the Northern Territory of Australia where melioidosis is endemic. *Burkholderia recA* PCR negative samples were further characterized using 16s *rRNA* sequencing for species identification. Phylogenetic analysis demonstrated that over 70% of the bacterial isolates were identified as *B*. *ubonensis* indicating that this species is common in the soil where *B*. *pseudomallei* is endemic. Bayesian phylogenetic analysis reveals many novel branches within the *B*. *cepacia* complex, one novel *B*. *oklahomensis*-like species, and one novel branch containing one isolate that is distinct from all other samples on the phylogenetic tree. During the analysis with *recA* sequencing, we discovered 2 single nucleotide polymorphisms in the reverse priming region of *B*. *oklahomensis*. A degenerate primer was developed and is proposed for future use. We conclude that the *recA* sequencing technique is an effective tool to classify *Burkholderia* and identify soil organisms in a melioidosis endemic area.

## Author Summary

*Burkholderia* is a widespread genus of bacteria that contains over 60 species. The species within *Burkholderia* range from environmentally important, such as isolates that breakdown pollutants, to human pathogens. *Burkholderia pseudomallei*, the causative agent of melioidosis, is endemic to Northern Australia and is a major public health concern in that region. We characterized 152 unknown environmental isolates using *recA* sequencing to identify the presence of *Burkholderia* bacteria in the region. The majority of bacteria identified in this study belonged to the *B*. *ubonensis* species and various species were found to inhabit the same environmental sample as the human pathogen, *B*. *pseudomallei*. The role of *B*. *ubonensis* and other *Burkholderia* species identified in this endemic region and how they interact with *B*. *pseudomallei* are important questions to address in order to understand the evolution of this important human pathogen. While they are occupying a similar geographic range, they must be exist in different niche in order to remain distinct species.

## Introduction

The *Burkholderia* genus contains a large number of species, with currently over 60 species identified [1]. Recently, the division of the genus *Burkholderia* has been proposed that breaks the genus into two genera. *Burkholderia* includes the clinical and phytopathogenic species whereas *Paraburkholderia* includes environmental species [2]. This *Burkholderia* genus occupies a large range of environments including soil, plants, rhizospheres, mammalian hosts, and water [3]. The ability of *Burkholderia* to occupy various ecological niches is undoubtedly due to the large genome size with up to three chromosomes documented in some *B*. *cepacia* complex organisms [4], and two chromosomes in five members of the Pseudomallei group including *B*. *pseudomallei*, *B*. *mallei*, *B*. *thailandensis*, proposed *B*. *humptydooensis*, and *B*. *oklahomensis*. Soil is a common habitat for *Burkholderia* bacteria and microbiologists are only beginning to uncover the complex nature of microbial communities in soil [5]. Continued sampling of the soil has uncovered many novel *Burkholderia* species in recent years [6–9]. This discovery of novel species across a wide geographic range has caused scientists to begin studying the role of *Burkholderia* in the soil community, and interactions with co-inhabitants [10].

One such interaction was found between *Burkholderia* in the soil in a study that showed *B*. *ubonensis* to have antaqonistic activity against *B*. *pseudomallei* in Papa New Guinea [11]. Recently, the geographic ranges of two species, *B*. *ubonensis* and *B*. *thailandensis*, were extended to a new area, Australia [12]. This type of discovery further supports the notion that *Burkholderia* isolates are very widespread, even at the species level. The identification of novel species within the melioidosis endemic region of Northern Australia is important to clinicians as they are confronted with patients presenting with a diverse variety of symptoms requiring consideration of and laboratory investigation for melioidosis.

Identifying and characterizing novel species can be a cumbersome task. The current standard for characterizing species in *Burkholderia* spp. includes biochemical testing, whole genome DNA-DNA hybridization, 16S *rRNA* sequencing, *recA* sequencing, antibiotic sensitivity, fatty acid methyl ester analysis and multi-locus sequence typing. The ability to rapidly and accurately identify novel *Burkholderia* species within the genus is crucial to understanding the complexity of this genus as well as its evolution. The *recA* gene has been shown to be particularly helpful in identifying the 17 species within the *Burkholderia cepacia* complex [13]. In 2005, *Burkholderia* species specific *recA* primers were developed that amplify all *Burkholderia* species and subsequent phylogenetic analysis distinguished closely related species [13]. This typing scheme has also been successful in identifying novel *Burkholderia* species associated with the rhizosphere using a cultivation independent method [14].

In our study, we applied the *recA* sequence typing scheme to unknown environmental samples taken from a melioidosis endemic region of Australia to determine the presence and diversity of *B*. *pseudomallei* near neighbors. During our analysis we modified the reverse primer of the *recA* assay to include additional species that have been identified and characterized since the assay was created in 2005.

## Methods

### Bacterial isolation and DNA preparation

From 86 environmental samples a total of 152 bacterial isolates were identifies as potential *B*. *pseudomallei* near neighbors. Environmental sampling was conducted in the Northern Territory of Australia concentrating on Darwin and the surrounding areas and included both water and soil sampling. Soil sampling produced 89 bacterial isolates and water sampling produced 63 bacterial isolates used in this study. water samples were collected from bores, tanks, wells, and ground water. Each sample was filtered through a 0.22-μm filter (Millipore Corporation, Bedford, MA, USA). The filters were inoculated into Ashdown’s broth at 37°C and subcultured on Ashdown’s agar (ASA) (Oxoid, Melbourne, Victoria, Australia) on day 2 and day 7 and incubated at 37°C until colonies similar to *Burkholderia* were observed. Soil samples were collected at two depths; 10cm and 30cm. For each sample, 20 grams of soil was mixed with 20mL of water and incubated at 37°C 48 hrs. A volume of 10mL of supernatant was inoculated into Ashdown’s broth and incubated at 37°C. Samples were subcultured onto Ashdown’s agar on day 2 and day 7 and incubated at 37°C until colonies similar to *Burkholderia* were observed. Each bacterial colony with a morphotype suspected to be *Burkholderia* was isolated in pure culture, given MSMB (Menzies School of Health Research Miscellaneous Bacteria) designations, and stored in Luria-Bertani broth plus 20% glycerol at -80°C (Difco, USA). Isolated colonies were grown on Tryptic Soy Agar (TSA) at 37°C for DNA preparation. DNA was extracted according to the manufacturer’s instructions using the Wizard Genomic DNA Purification Kit (Promega, Madison, WI, USA).

### PCR analysis

Samples were genotyped using several published genomic target assays. Assays such as TTS1 [15], targeting the *B*. *pseudomallei* Type III secretion cluster; BTFC [16], targeting the *B*. *thailandensis*-like flagella and chemotaxis cluster; and YLF[16], targeting the *Yersinia*-like fimbrial gene cluster were used to identify *B*. *pseudomallei* strains. *cheB [16]*, targeting the *B*. *thailandensis* homolog to BTFC was used to identify *B*. *thailandensis* samples. LPS A, B, and B2, targeting the lipopolysaccharide (LPS) genes, were used to identify LPS genotypes across species (26). Real-time PCR assays were conducted in a 384-well plate in 10μL reactions containing 1 x SYBR-Green master mix (Applied Biosystems, USA), 0.3μM of each PCR primer, and 1.0ng of DNA. The real-time PCR assay was performed on a 7900HT Sequence Detection System (Applied Biosystems, USA). A total of 40 cycles were performed that included two steps: denaturation at 95°C for 15s and annealing/extension at 60°C for 30s. We also generated a dissociation curve from 95°C to 60°C to analyze the melt temperatures of the PCR products.

Previously published *recA* sequencing primers, BUR3 and BUR4, were used to analyze the unknown samples[13]. Universal 16s *rRNA* primers were used for all isolates that were BUR3-BUR4 negative[17]. PCR assays were conducted in a 96-well plate in 20μL reactions. Each PCR reaction contained the following: 1 U of Platinum Taq polymerase (Life Technologies, USA), 1x PCR Buffer (Life Technologies, USA), 1.5mM MgCl_2_, 0.25mM dNTPs, 24% (w/v) Betaine, and 0.2μM of each primer. A modified PCR protocol, ‘slow-down PCR’, specifically developed for high G-C content organisms was used for these samples [18,19]. The following modification to the cycling protocol was made: the annealing temperature started at 65°C and the extension time lengthened to 3 minutes.

### Sequence analysis

*recA* PCR products were sequenced according to the published methods by Payne *et al* [13]. All BUR3-BUR4 negative samples were sequenced using universal 16S *rRNA* primers targeting the hypervariable region V3 and V4 [20]. Sequencing reactions were prepared using Big Dye Terminator v3.1 in 1/8 reactions according to the manufacturer’s instructions and analyzed on a 3130 genetic analyzer (Life Technologies, USA). Consensus sequences were generated by aligning raw sequences from both strands using DNASTAR software (DNASTAR, Inc. Madison, WI). Species identity was established by phylogenetic analysis for *recA* sequences and through basic local alignment sequence tool (BLAST) at the National Center for Biotechnology Information (http://www.ncbi.nlm.nih.gov/) for 16S *rRNA* sequences.

### Phylogenetic analysis of *Burkholderia recA*

Sequence alignments of all unknown samples and reference strains were carried out in Clustal X [21]. J Model Test 2 was run to determine the best nucleotide substitution model for Bayesian analysis [22]. We used 335bp of the *recA* sequence from 196 taxa, 115 taxa from this study, with the Bayesian analysis in Mr. Bayes v3. Parameters for Mr. Bayes are as follows: 6 substitution types, which allows for all rates of substitution to different, subject to the constraint of time-reversibility (a generalized time reversible model); haploid; gamma distribution for among-site rate variation; 11,000 generations for Markov Chain Monte Carlo (MCMC) analysis; Sample the MCMC analysis every 1000 generations; and 0.1% of samples will be discarded when convergence diagnostics are calculated. All other parameters in Mr. Bayes were left to the default settings. Phylogenetic trees were created in FigTree v1.3.1. The tree was rooted with *recA* sequence from *Burkholderia spp*. R701.

### Geographic distribution of *Burkholderia* in the Northern Territory

GPS data acquired during the environmental sampling and Arc Map10 were used to map samples and determine any phylogeographic patterns.

### *recA* assay development

Alignments of 31 available *recA* sequences from different *Burkholderia* species were compared to identify a specific region that can be used to develop a new reverse primer. Several reverse primers were identified and tested using the PCR method described above against a panel of 83 *Burkholderia* isolates and 13 species outside the *Burkholderia* genus.

### Nucleotide sequence accession numbers

*recA* nucleotide sequences were determined for 115 *Burkholderia* isolates and submitted to GenBank under the following accession numbers; KF204451 through KF204565.

## Results

### Bacterial isolation

**Fig 1:**
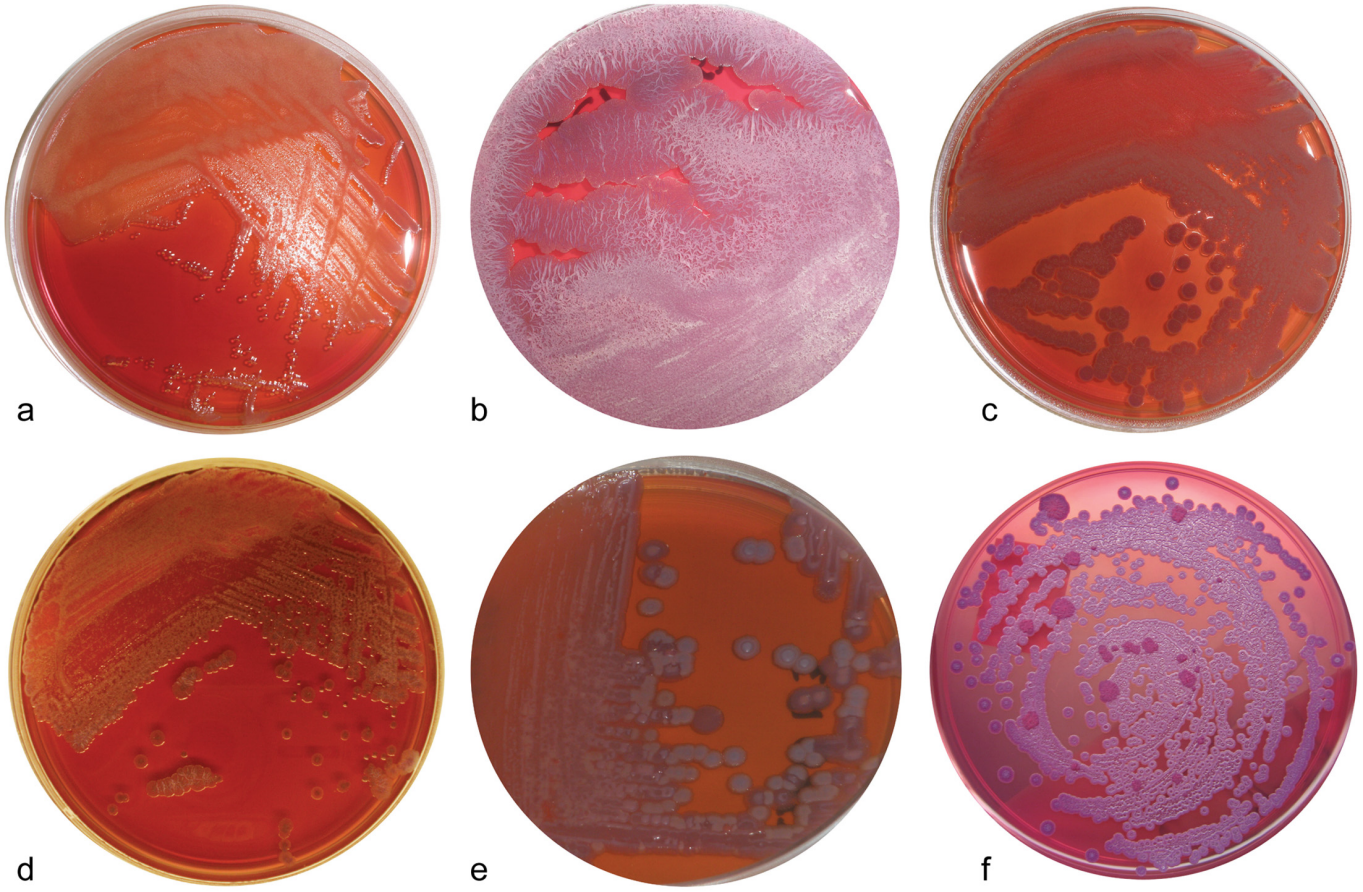
Colony morphology of MSMB isolates on Ashdown’s Agar (ASA) compared to *B*. *pseudomallei* MSHR305. ASA is considered a selective growth medium for *Burkholderia pseudomallei*. In this study, at least 20 different species demonstrated the ability to grow on ASA including multiple *Burkholderia* speices as well as *Ralstonia*, *Cupriavidus*, and *Panoraea*. Many different colony morphologies were observed during this study as evident in the pictures (a) proposed *B*. *humptydooensis*MSMB43, (b) unknown *Burkholderia spp*. MSMB 175, (c) *B*. *multivorans* MSMB105, (d) *B*. *thailandensis* MSMB60, (e) *B*. *ubonensis* MSMB153, (f) *B*. *pseudomallei* MSHR 305. Each strain was grown on ASA for 72 hours at 37°C aerobically.

A total of 152 soil isolates from this study successfully grew on both ASA and TSA at 37°C. ASA is classically known as a selective medium for *B*. *pseudomallei* [23,24]. In this study, we found that many different genera of bacteria were able to grow on this medium including *Ralstonia spp*, *Pandoraea spp*, *Cupriavidus spp*, and *Delftia spp*. Colony morphologies observed on ASA agar in this study ranged from light purple, large, dry and wrinkled to very dark purple, shiny, small, smooth colonies as shown in Fig 1. Although morphologies varied among species, it is not sufficient to identify the species of an isolate based solely on its ability to grow on ASA and colony morphology. S1 Fig highlights colony morphologies of various species isolated during this study.

### PCR and species identification

Isolates used in this study were tested against previously published assays to identify their genetic characteristics. These particular genes have been used previously to characterize subpopulations of *B*. *pseudomallei* and they are also known to be horizontally transferred within among Burkholderia species, making their presence/absence of interest in these close relatives that live in the same environmental locations. Only two isolates, MSMB262 and MSMB313, were positive for Type III Secretion System (TTS) SYBR assay [15] signifying that these isolates are *B*. *pseudomallei*. These results were later confirmed using *recA* sequencing [13]. Isolates were also tested against other assays including BTFC/YLF [16], *cheB* [25], *bimA* [26], LPS [27]. Two isolates were found to be *cheB* positive which indicates that these isolates are *B*. *thailandensis* (25). We were able to verify these results using *recA* sequencing that confirmed the samples were *B*. *thailandensis*. Some interesting findings from the study include an unclassified *Burkholderia* species MSMB264 that is positive for the BTFC assay. MSMB264 may have a similar gene to BTFC that will need to be investigated further. All proposed *B*. *humptydooensis* organisms were found to contain the *B*. *mallei* version of *bimA*; one strain of *Ralstonia*, MSMB 132, also contained this *bimA* type [28]. These results will require additional analysis to determine the gene content. Four isolates were found to be positive for LPS type A; the two *B*. *pseudomallei* and the two *B*. *thailandensis* isolates. Thirteen isolates were positive for LPS type B; 11 *B*. *ubonensis* isolates and two unknown *B*. *cepacia* complex isolates. Four proposed *B*. *humptydooensis* isolates were found to be positive for LPS type B2. These PCR results are summarized in Table 1 [28].

**Table 1:** Comparison of environmental species identified in the Darwin region of Australia.

Species	Number of Isolates	Number of Sites^a^	Soil/H_2_O	Occurs with *B*. *pseudomallei* ^*b*^	*recA* BUR3-BUR4	BTFC	YLF	*cheB*	*bimA*_bp_	*bimA*_bm_	LPSA	LPSB	LPSB2
***B*. *ubonensis***	**81**	**34**	**60/21**	**20**	**81**	**-**	**-**	**-**	**-**	**-**	**-**	**11**	**-**
**Unknown *B*. *cepacia* complex**	**16**	**13**	**10/6**	**5**	**16**	**-**	**-**	**-**	**-**	**-**	**-**	**2**	**-**
***B*. *humptydooensis***	**6**	**2**	**0/6**	**1**	**6**	**-**	**-**	**-**	**-**	**6**	**-**	**-**	**5**
***B*. *multivorans***	**3**	**2**	**2/1**	**-**	**3**	**-**	**-**	**-**	**-**	**-**	**-**	**-**	**-**
***B*. *pseudomallei***	**2**	**2**	**2/0**	**2**	**2**	**2**	**-**	**-**	**2**	**-**	**2**	**-**	**-**
***B*. *cenocepacia***	**2**	**1**	**0/2**	**-**	**2**	**-**	**-**	**-**	**-**	**-**	**-**	**-**	**-**
***B*. *thailandensis***	**2**	**1**	**2/0**	**-**	**2**	**-**	**-**	**2**	**-**	**-**	**2**	**-**	**-**
***B*. *seminalis***	**1**	**1**	**1/0**	**-**	**1**	**-**	**-**	**-**	**-**	**-**	**-**	**-**	**-**
***B*. *oklahomensis*-like**	**1**	**1**	**1/0**	**-**	**1**	**1**	**1**	**-**	**-**	**-**	**-**	**-**	**-**
**Unknown *B*. *spp*.**	**1**	**1**	**1/0**	**-**	**1**	**1**	**-**	**-**	**-**	**-**	**-**	**-**	**-**
***Ralstonia***	**12**	**7**	**0/12**	**-**	**-**	**-**	**-**	**-**	**-**	**1**	**-**	**-**	**-**
***Cupriavidus***	**7**	**7**	**1/6**	**1**	**-**	**-**	**-**	**-**	**-**	**-**	**-**	**-**	**-**
***Pandoraea***	**5**	**2**	**5/0**	**1**	**-**	**-**	**-**	**-**	**-**	**-**	**-**	**-**	**-**
***Delftia***	**3**	**3**	**2/1**	**-**	**1**	**-**	**-**	**-**	**-**	**-**	**-**	**-**	**-**
***Pigmentiphaga***	**2**	**1**	**0/2**	**-**	**1**	**-**	**-**	**-**	**-**	**-**	**-**	**-**	**-**
***Achromobacter***	**2**	**2**	**0/2**	**-**	**-**	**-**	**-**	**-**	**-**	**-**	**-**	**-**	**-**
***Bacillus***	**1**	**1**	**1/0**	**-**	**-**	**-**	**-**	**-**	**-**	**-**	**-**	**-**	**-**
***Comomonas***	**1**	**1**	**0/1**	**-**	**-**	**-**	**-**	**-**	**-**	**-**	**-**	**-**	**-**
***Staphylococcus***	**1**	**1**	**0/1**	**-**	**-**	**-**	**-**	**-**	**-**	**-**	**-**	**-**	**-**
***Stenotrophomonas***	**1**	**1**	**0/1**	**-**	**-**	**-**	**-**	**-**	**-**	**-**	**-**	**-**	**-**
**Unknown**	**2**	**2**	**1/1**	**-**	**-**	**-**	**-**	**-**	**-**	**-**	**-**	**-**	**-**

(-) indicates that no *B*. *pseudomallei* was found to occur with a species or that the molecular assay was negative for that target.

^a^ Site refers to the GPS location from which soil or water samples were taken.

^b^ *B*. *pseudomallei* were recovered from the same soil or water sample as the isolate.

### Phylogenetic analysis of *Burkholderia recA*

**Fig 2:**
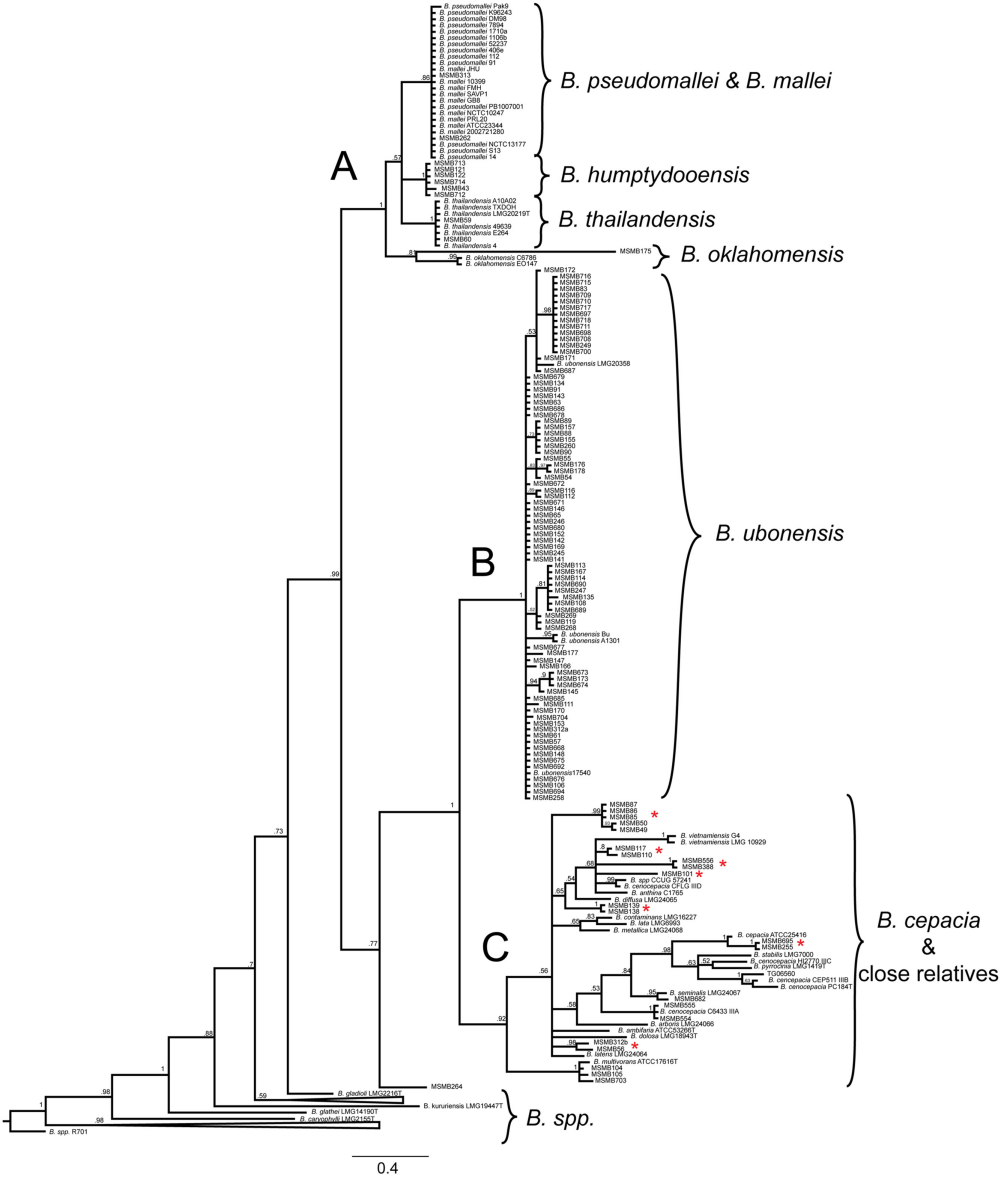
Phylogenetic analysis of environmental *Burkholderia* spp. sampled from soil in the Northern Territory of Australia. A 335-bp region of the *recA* gene was sequenced and analyzed using Bayesian methods. The phylogenetic tree is the consensus of 19,000 trees and 11 million generations using the nucleotide substitution model GTR+G. Species and isolate names are shown for reference sequences. Clade A highlights the Pseudomallei group of *Burkholderia* species where we identified several isolates of proposed *B*. *humptydooensis* and *B*. *thailandensis*. Clade B highlights the large number of *B*. *ubonensis* isolates identified in the northern territory of Australia during this study. Clade C is the *B*. *cepacia* complex outside of *B*. *ubonensis*. Posterior probabilities are assigned at branch nodes (1 = 100%). Scale bar signifies nucleotide substitutions per site. Clade C demonstrates the diversity found within the *B*. *cepacia* complex. Several novel nodes, marked with an asterisk, are evident in this phylogenetic tree which may represent novel species.

Bayesian analysis conducted using 196 taxa on a 335bp region of the *recA* gene reveals great diversity within the *Burkholderia* genus. A large proportion (72%) of the MSMB isolates from this study grouped with *B*. *ubonensis* as shown in Table 1 and Fig 2. Several isolates grouped within the previously identified proposed species *B*. *humptydooensis* isolates including MSMB121, MSMB122, MSMB712, MSMB713, and MSMB714 [28]. One singleton, MSMB175, appears to be closely related to the *B*. *oklahomensis* species based upon the *recA* gene. Another singleton, MSMB264 is separated from all classically defined groups. Clade C of Fig 2 shows the substantial diversity within the *B*. *cepacia* complex. In this study alone, at least 7 new *B*. *cepacia* complex groups were identified in the *recA* phylogenetic tree that may represent novel species.

### 16s *rRNA* analysis

Using universal 16s *rRNA* primers, we were able to identify 37 isolates that were negative to the BUR3-BUR4 *recA* assay. BLAST cutoffs for identifying the genus were set at 99%. The three most prevalent genera identified outside of the *Burkholderia* genus were *Ralstonia*, *Cupriavidus*, and *Pandoraea*. Other species identified were *Achromobacter*, *Bacillus*, *Comamonas*, *Delftia*, *Pigmentiphaga*, *Staphylococcus*, and *Stenotrophomonas*. Two isolates in this study were unable to be identified by *recA* and 16s *rRNA* sequencing. The sequence quality for each of these isolates was low and no conclusions about their genus or species could be made.

### Geographic distribution of *Burkholderia* in the Northern Territory

**Fig 3:**
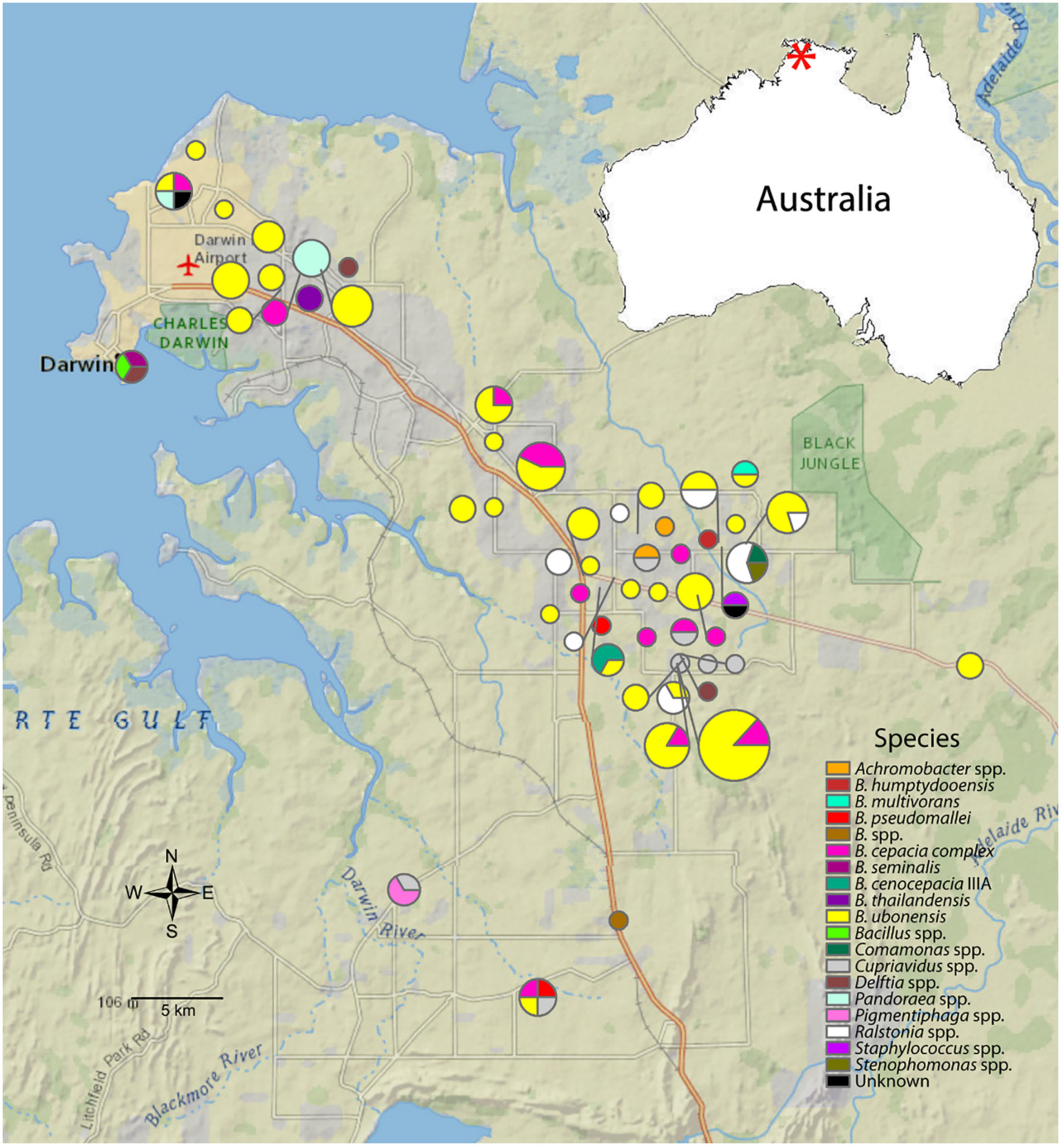
Geographical distribution of isolates from this study. Circle size represents the number of isolates found at a specific sampling site and the color represents different species as determined by *recA* or 16s *rRNA* sequencing. Division of circles indicates that multiple species were found at a site. *B*. *ubonensis* is commonly found throughout Darwin and the surrounding areas. Several species were found in the same soil sample as *B*. *pseudomallei* including *B*. *ubonensis*, several *B*. *cepacia* complex species, proposed *B*. *humptydooensis*, *Pandoraea*, and *Cupriavidus*.

Longitudinal and latitudinal data collected during soil sampling was used to generate a map of the Northern Territory region to look for patterns in the occurrence of *B*. *pseudomallei* near neighbor isolates (Fig 3). *B*. *thailandensis-like* MSMB43, a potential new species being proposed as *B*. *humptydooensis* and collected in Humpty Doo is more than 1200 kilometers north from the new isolates MSMB712, MSMB713, and MSMB714 collected in the Tennant creek area of central Australia [28,29]. *B*. *ubonensis* is widespread throughout Darwin and the surrounding areas and is found in the same soil samples as other *Burkholderia* species including other *B*. *cepacia* complex organisms as well as *Burkholderia* near neighbors such as *Pandoraea*, *Cupriavidus*, and *Ralstonia*. *B*. *ubonensis*, *B*. *cepacia* complex, and *B*. *multivorans* samples were isolated from both water and soil environments (see Table 1). Six separate species including, *B*. *ubonensis*, *B*. *cepacia* complex, proposed *B*. *humptydooensis*, *Cupriavidus* spp., and *Pandoraea* spp, were found in the same environmental sample as the human pathogen *B*. *pseudomallei* [28]. This indicates that various species of *Burkholderia* are occupying the same environmental niche and may be sharing genes through lateral gene transfer.

### *recA* assay

Previously published primers, BUR3 and BUR4, were used to identify all of the *Burkholderia* isolates in this study [13]. During the testing we noticed that two other genera, *Delftia* and *Pandoraea*, were also amplified with this primer set. Upon further investigation, BLAST analysis revealed a 100% match in the genome of *Delftia* spp. to BUR3 and BUR4. During the study, we found that BUR3 and BUR 4 did not amplify certain strains of *B*. *oklahomensis*. Upon *in silico* analysis, we observed two single nucleotide polymorphisms in the reverse priming region in *B*. *oklahomensis*. Nucleotide alignments of *recA* for 16 different species within the genus *Burkholderia* were used to develop the new reverse primer. This new degenerate primer, BUR5 5’-CGATCATGTCGATCGARC-3’, is located 11 base pairs upstream of the BUR4 primer. The new BUR3-BUR5 *recA* fragment is 376bp in length. The new primer was validated against a panel of 98 isolates encompassing at least 19 different species within the *Burkholderia* genus and 15 species outside of *Burkholderia*. The 19 inclusion species were: *B*. *pseudomallei*, *B*. *mallei*, *B*. *thailandensis*, *B*. *humptydooensis*, *B*. *oklahomensis*, *B*. *oklahomensis*-like, *B*. *ubonensis*, *B*. *ambifaria*, *B*. *multivorans*, *B*. *vietnamiensis*, *B*. *fungorum*, *B*. *glumae*, *B*. *cepacia*, *B*. *xenovorans*, *B*. *dolosa*, *B*. *gladioli*, *B*. *cepacia* complex, and 2 *B*. spp. The 15 exclusionary test species were: *Staphylococcus* spp., *S*. *aureus*, *Yersinia pestis*, *Pseudomonas aeruginosa*, *P*. *fluorescens*, *Streptococcus mitis*, *S*. *salivarius*, *S*. *viridans*, *Bacillus* spp., *Brucella abortus*, *Brucella suis*, *Francisella tularensis*, *Cupriavidus* spp., *Ralstonia* spp., and *Escherichia coli*. None of the exclusionary text species amplified with the PCR conditions described in the methods section.

## Discussion

In this study, we used a *Burkholderia* specific *recA* genotyping scheme to identify unknown environmental isolates from Northern Australia that resemble *B*. *pseudomallei* morphology on ASA. Many *Burkholderia* species throughout the *B*. *cepacia* complex and Pseudomallei group were identified during this study. A majority of species identified belong to the *B*. *ubonensis* species based upon *recA* phylogenetics. *B*. *ubonensis* was only recently discovered in Australian soil [12] and our study further supports that this species is found in great abundance in both soil and water throughout the Darwin region of Australia. *B*. *ubonensis* was recently found to have antagonistic activity against *B*.*pseudomallei* by an unspecified bacteriocin or bacteriocin-like inhibitory substance in Papua New Guinea [11]. An investigation into the *B*. *ubonensis* isolates from this study would determine if these isolates contain the same ability to inhibit *B*. *pseudomallei* growth as found in Papua New Guinea and further develop our understanding the role of *B*. *ubonensis* in the environment. Information from this study and the prevalence of *Burkholderia* species within endemic regions will continue to advance our knowledge of the bacterial composition of the natural environment. This will allow scientists to develop more specific molecular assays for targeting such agents as *B*. *pseudomallei* by understanding the background and potential for false positive results. Continued sampling efforts should be conducted to obtain an even deeper understanding of the geographic distribution of *Burkholderia* in this region. It is important to note that the methods in this study included using selective media, ASA, to isolate the *B*. *pseudomallei* near neighbors. There is a potential some novel near neighbors were not discovered due to the use of this media.

The phylogenetic analysis used in this study provides a way to quickly assess the species of an unknown environmental sample from the Northern Territory of Australia. Isolates that are novel based upon *recA* phylogenetic analysis can be sent for whole genome sequencing at a relatively low cost to evaluate the true genetic relatedness of the isolate to previously characterized species. With next generation sequencing costs continuing to decline, this method offers a way to screen a large number of isolates and then send only the unique isolates for whole genome sequencing. To find additional diversity within the *Burkholderia* genus, samples from other melioidosis-endemic areas such as Thailand and other Southeast Asian countries should be evaluated. That data would provide information on the presence of *B*. *pseudomallei* near neighbor organisms in levels that are similar or different to what we have found in the Darwin region of Northern Australia.

Many novel branches were identified in the phylogenetic analysis of this study. A majority of novel branches were found in the *B*. *cepacia* complex, a complex of bacteria that is important to the study of cystic fibrosis. These novel branches and the isolates within them need to be further investigated to determine their true uniqueness within the complex. Two interesting isolates were identified in this study, MSMB175 and MSMB264. MSMB175 is an isolate that groups within the *B*. *oklahomensis* branch. *B*. *oklahomensis* has never previously been documented in Australia. The genomes from the Australian *B*. *oklahomensis*-like isolate and the type strain of *B*. *oklahomensis* should be compared to determine how much of the core and accessory genome is shared between these isolates. Samples from this study were isolated from both soil and water: two very complex environments. The microbial diversity within a soil niche, how soil composition impacts microbial populations and how speciation occurs are areas of research that are not well understood. With recent developments in metagenomics through next generation sequencing, these questions are now beginning to be addressed. From recent studies we know that the local environment substantially impacts where the human pathogen *B*. *pseudomallei* is found, with likely important regional-specific factors for different landscapes globally [30,31]. Understanding how speciation occurs in the soil environment would be beneficial to our knowledge of the evolution of pathogens and acquisition of virulence factors. In this study, we found a diverse group of *Burkholderia* species inhabiting the Darwin region including the human pathogen, *B*. *pseudomallei*. The identification of 9 potentially novel *Burkholderia* species within this endemic melioidosis region opens up many more questions about the behavior of this genus in the environment. We found six instances where multiple species of *Burkholderia* were cultured from the same soil sample. Given the genomic plasticity of the *Burkholderia* genome and the understanding that *Burkholderia* organisms are found in the same environmental sample lends support to the idea that *Burkholderia* species may freely exchange genetic material between each other in the environment [32,33]. One probable example of this is demonstrated in the lipopolysaccharide genes. *B*. *ubonensis*, *B*. *thailandensis*, proposed *B*. *humptydooensis*, and other *Burkholderia* species are known to express O-antigen portions of the lipopolysaccharide that are highly similar to *B*. *pseudomallei* [28,34]. Through the analysis of genomes of *Burkholderia* and near neighbor isolates found occupying the same niche, we can begin to understand the role of lateral gene transfer among these organisms.

## Supporting Information

S1 Fig Morphology of MSMB Isolates. (PDF)

## References

[1] Vial L, Chapalain A, Groleau M-C, Déziel E (2011) The various lifestyles of the *Burkholderia cepacia* complex species: a tribute to adaptation. Environ Microbiol 13: 1–12. 10.1111/j.1462-2920.2010.02343.x 20880095

[2] Sawana A, Adeolu M, Gupta RS (2014) Molecular signatures and phylogenomic analysis of the genus *Burkholderia*: proposal for division of this genus into the emended genus *Burkholderia* containing pathogenic organisms and a new genus *Paraburkholderia* gen. nov. harboring environmental species. Front Genet 5. 10.3389/fgene.2014.00429 PMC4271702 25566316

[3] Compant S, Nowak J, Coenye T, Clément C, Ait Barka E (2008) Diversity and occurrence of *Burkholderia* spp. in the natural environment. FEMS Microbiol Rev 32: 607–626. 10.1111/j.1574-6976.2008.00113.x 18422616

[4] Agnoli K, Schwager S, Uehlinger S, Vergunst A, Viteri DF, et al (2012) Exposing the third chromosome of *Burkholderia cepacia* complex strains as a virulence plasmid. Molecular Microbiology 83: 362–378. 10.1111/j.1365-2958.2011.07937.x 22171913

[5] Lauber CL, Hamady M, Knight R, Fierer N (2009) Pyrosequencing-based assessment of soil pH as a predictor of soil bacterial community structure at the continental scale. Appl Environ Microbiol 75: 5111–5120. 10.1128/AEM.00335-09 19502440 PMC2725504

[6] Howieson JG, De Meyer SE, Vivas-Marfisi A, Ratnayake S, Ardley JK, et al Novel *Burkholderia* bacteria isolated from *Lebeckia ambigua–*A perennial suffrutescent legume of the fynbos. Soil Biol Biochem 60: 55–64.

[7] Vanlaere E, LiPuma JJ, Baldwin A, Henry D, De Brandt E, et al (2008) *Burkholderia latens* sp. nov., *Burkholderia diffusa* sp. nov., *Burkholderia arboris* sp. nov., *Burkholderia seminalis* sp. nov. and *Burkholderia metallica* sp. nov., novel species within the *Burkholderia cepacia* complex. Int J Syst Evol Microbiol 58: 1580–1590. 10.1099/ijs.0.65634-0 18599699

[8] Srinivasan S, Kim J, Kang S-R, Jheong W-H, Lee S-S (2013) *Burkholderia humi* sp. nov., isolated from peat soil. Curr Microbiol 66: 300–305. 10.1007/s00284-012-0270-9 23196702

[9] Zhu H, Guo J, Chen M, Feng G, Yao Q (2012) *Burkholderia dabaoshanensis* sp. nov., a heavy-metal-tolerant bacteria isolated from Dabaoshan mining area soil in China. PLoS One 7: e50225 10.1371/journal.pone.0050225 23226514 PMC3514224

[10] Coenye T, Vandamme P (2003) Diversity and significance of *Burkholderia* species occupying diverse ecological niches. Environ Microbiol 5: 719–729. 12919407 10.1046/j.1462-2920.2003.00471.x

[11] Marshall K, Shakya S, Greenhill AR, Padilla G, Baker A, et al (2010) Antibiosis of *Burkholderia ubonensis* against *Burkholderia pseudomallei*, the causative agent for melioidosis. Southeast Asian J Trop Med Public Health 41: 904–912. 21073065

[12] Levy A, Merritt AJ, Aravena-Roman M, Hodge MM, Inglis TJJ (2008) Expanded range of *Burkholderia* species in Australia. Am J Trop Med Hyg 78: 599–604. 18385355

[13] Payne GW, Vandamme P, Morgan SH, LiPuma JJ, Coenye T, et al (2005) Development of a *recA* gene-based identification approach for the entire *Burkholderia* genus. Appl Environ Microbiol 71: 3917–3927. 16000805 10.1128/AEM.71.7.3917-3927.2005 PMC1169057

[14] Payne GW, Ramette A, Rose HL, Weightman AJ, Jones TH, et al (2006) Application of a *recA* gene-based identification approach to the maize rhizosphere reveals novel diversity in *Burkholderia* species. FEMS Microbiol Lett 259: 126–132. 16684112 10.1111/j.1574-6968.2006.00257.x

[15] Novak R, Glass M, Gee J, Gal D, Mayo M, et al (2006) Development and evaluation of a real-time PCR assay targeting the type III secretion system of *Burkholderia pseudomallei* . J Clin Microbiol 44: 85–90. 16390953 10.1128/JCM.44.1.85-90.2006 PMC1351940

[16] Tuanyok A, Auerbach R, Brettin T, Bruce D, Munk A, et al (2007) A horizontal gene transfer event defines two distinct groups within *Burkholderia pseudomallei* that have dissimilar geographic distributions. J Bacteriol 189: 9044–9049. 17933898 10.1128/JB.01264-07 PMC2168593

[17] Liu CM, Aziz M, Kachur S, Hsueh PR, Huang YT, et al (2012) BactQuant: an enhanced broad-coverage bacterial quantitative real-time PCR assay. BMC Microbiol 12: 56 10.1186/1471-2180-12-56 22510143 PMC3464140

[18] Bachmann HS, Siffert W, Frey UH (2003) Successful amplification of extremely GC-rich promoter regions using a novel 'slowdown PCR' technique. Pharmacogenetics 13: 759–766. 14646694 10.1097/00008571-200312000-00006

[19] Frey UH, Bachmann HS, Peters J, Siffert W (2008) PCR-amplification of GC-rich regions: 'slowdown PCR'. Nat Protoc 3: 1312–1317. 10.1038/nprot.2008.112 18714299

[20] Nadkarni MA, Martin FE, Jacques NA, Hunter N (2002) Determination of bacterial load by real-time PCR using a broad-range (universal) probe and primers set. Microbiology 148: 257–266. 11782518 10.1099/00221287-148-1-257

[21] Larkin MA, Blackshields G, Brown NP, Chenna R, McGettigan PA, et al (2007) Clustal W and Clustal X version 2.0. Bioinformatics 23: 2947–2948. 17846036 10.1093/bioinformatics/btm404

[22] Darriba D, Taboada GL, Doallo R, Posada D (2012) jModelTest 2: more models, new heuristics and parallel computing. Nat Meth 9: 772–772. 10.1038/nmeth.2109 PMC4594756 22847109

[23] Peacock SJ, Chieng G, Cheng AC, Dance DAB, Amornchai P, et al (2005) Comparison of Ashdown's medium, *Burkholderia cepacia* medium, and *Burkholderia pseudomallei* selective agar for clinical isolation of *Burkholderia pseudomallei* . J Clin Microbiol 43: 5359–5361. 16208018 10.1128/JCM.43.10.5359-5361.2005 PMC1248505

[24] Glass MB, Beesley CA, Wilkins PP, Hoffmaster AR (2009) Comparison of four selective media for the isolation of *Burkholderia mallei* and *Burkholderia pseudomallei* . Am J Trop Med Hyg 80: 1023–1028. 19478269

[25] Tuanyok A, Auerbach RK, Brettin TS, Bruce DC, Munk AC, et al (2007) A horizontal gene transfer event defines two distinct groups within Burkholderia pseudomallei that have dissimilar geographic distributions. Journal of bacteriology 189: 9044–9049. 17933898 10.1128/JB.01264-07 PMC2168593

[26] Ulrich RL, Ulrich MP, Schell MA, Kim HS, DeShazer D (2006) Development of a polymerase chain reaction assay for the specific identification of *Burkholderia mallei* and differentiation from *Burkholderia pseudomallei* and other closely related *Burkholderiaceae* . Diagn Microbiol Infect Dis 55: 37–45. 16546342 10.1016/j.diagmicrobio.2005.11.007

[27] Tuanyok A, Stone JK, Mayo M, Kaestli M, Gruendike J, et al (2012) The genetic and molecular basis of O-antigenic diversity in *Burkholderia pseudomallei* lipopolysaccharide. PLoS Negl Trop Dis 6: e1453 10.1371/journal.pntd.0001453 22235357 PMC3250505

[28] Currie BJ (2015) Melioidosis: evolving concepts in epidemiology, pathogenesis, and treatment. Semin Respir Crit Care Med 36: 111–125. 10.1055/s-0034-1398389 25643275

[29] Gee J, Glass M, Novak R, Gal D, Mayo M, et al (2008) Recovery of a *Burkholderia thailandensis*-like isolate from an Australian water source. BMC Microbiol 8: 54 10.1186/1471-2180-8-54 18384685 PMC2329625

[30] Kaestli M, Mayo M, Harrington G, Ward L, Watt F, et al (2009) Landscape changes influence the occurrence of the melioidosis bacterium *Burkholderia pseudomallei* in soil in northern Australia. PLoS Negl Trop Dis 3: e364 10.1371/journal.pntd.0000364 19156200 PMC2617783

[31] Kaestli M, Schmid M, Mayo M, Rothballer M, Harrington G, et al (2012) Out of the ground: aerial and exotic habitats of the melioidosis bacterium *Burkholderia pseudomallei* in grasses in Australia. Environ Microbiol 14: 2058–2070. 10.1111/j.1462-2920.2011.02671.x 22176696 PMC3319007

[32] Holden MTG, Titball RW, Peacock SJ, Cerdeno-Tarraga AM, Atkins T, et al (2004) Genomic plasticity of the causative agent of melioidosis, *Burkholderia pseudomallei* . PNAS 101: 14240–14245. 15377794 10.1073/pnas.0403302101 PMC521101

[33] Lessie TG, Hendrickson W, Manning BD, Devereux R (1996) Genomic complexity and plasticity of *Burkholderia cepacia* . FEMS Microbiol Lett 144: 117–128. 8900054 10.1111/j.1574-6968.1996.tb08517.x

[34] Stone J, Mayo M, Grasso S, Ginther J, Warrington S, et al (2012) Detection of *Burkholderia pseudomallei* O-antigen serotypes in near-neighbor species. BMC Microbiol 12: 1–8. 10.1186/1471-2180-12-1 23126230 PMC3541218

