# Supplementary material for: Identification of Burkholderia pseudomallei Near-Neighbor Species in the Northern Territory of Australia
Source: PLoS Negl Trop Dis. 2015 Jun 29;9(6):e0003892. doi: 10.1371/journal.pntd.0003892 (PMC4486726; doi:10.1371/journal.pntd.0003892)
Supplement: S1 Fig — (PDF) [file pntd.0003892.s001.pdf]

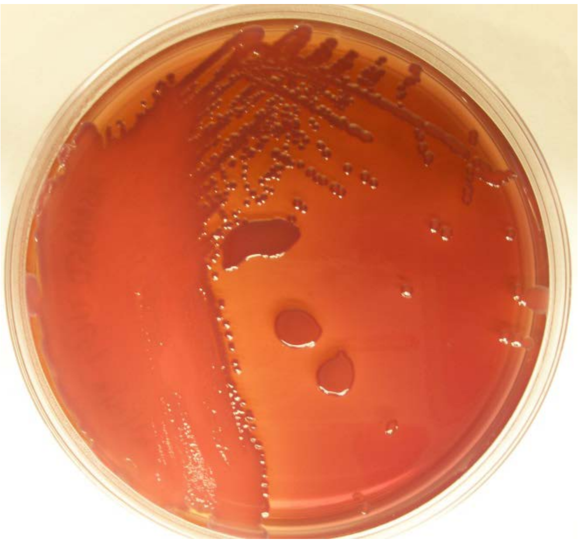

B. cepacia complex MSMB50

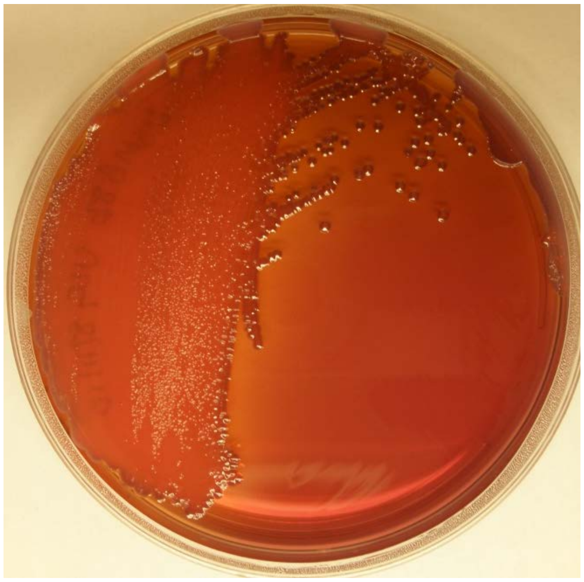

B. cepacia complex MSMB86

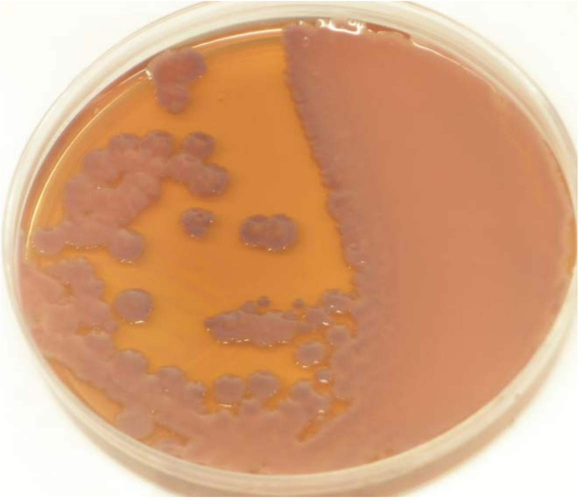

B. cepacia complex MSMB101

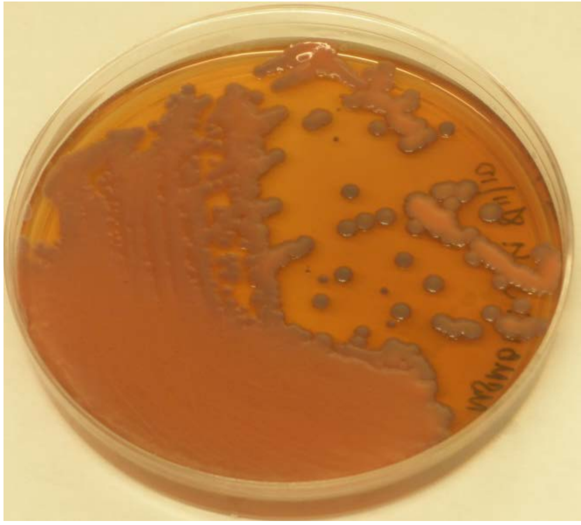

B. cepacia complex MSMB117

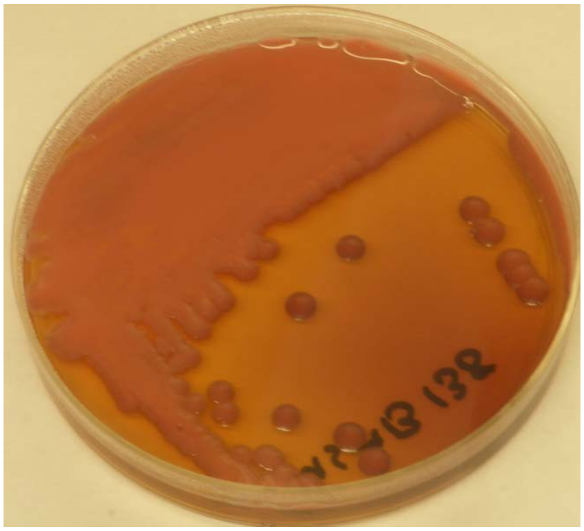

B. cepacia complex MSMB138

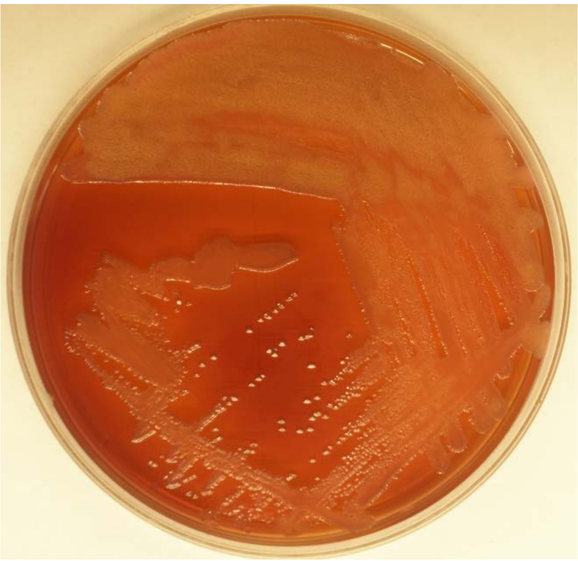

B. humptydooensis MSMB43

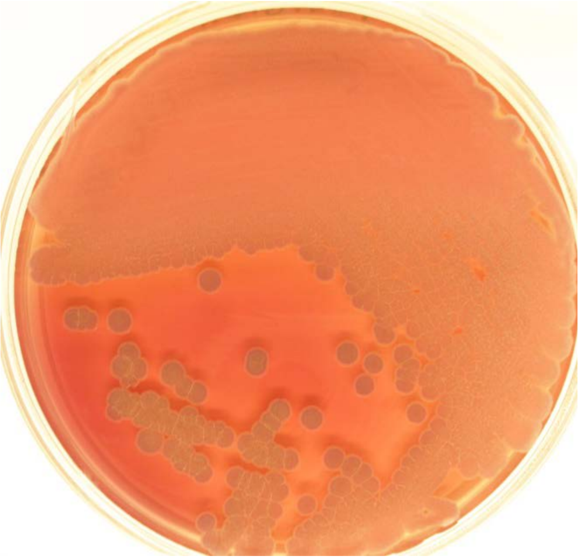

B. multivorans MSMB104

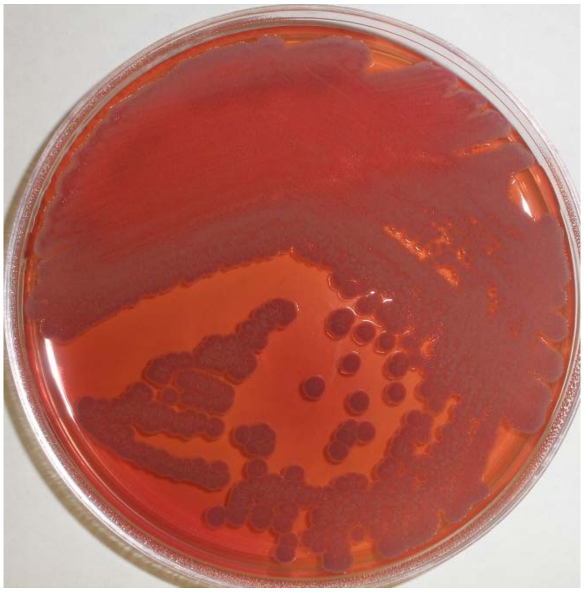

B. multivorans MSMB105

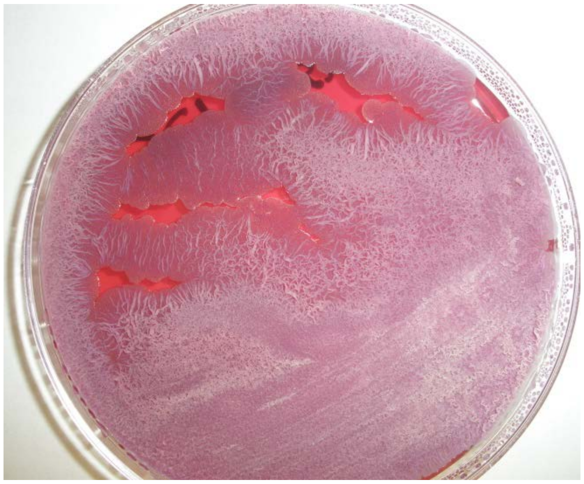

B. oklahomensis-like MSMB175

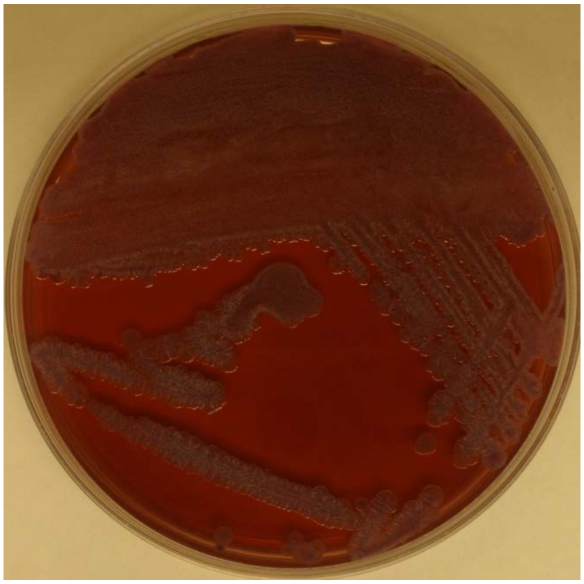

B. thailandensis MSMB59

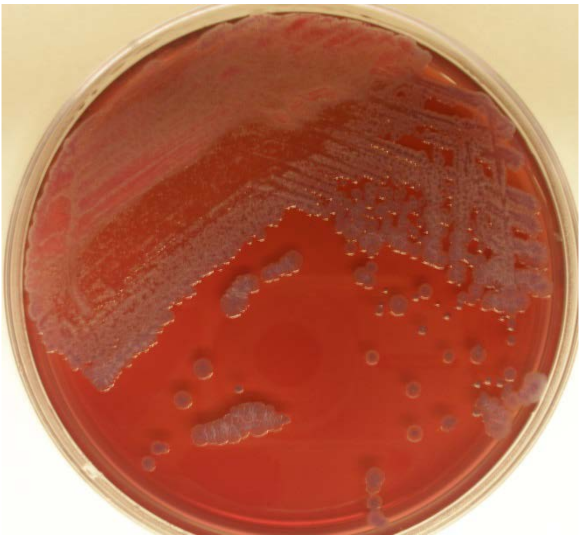

B. thailandensis MSMB60

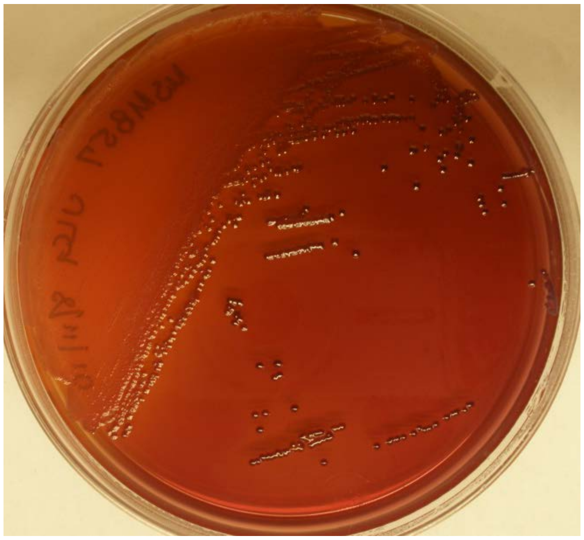

B. ubonensis MSMB57

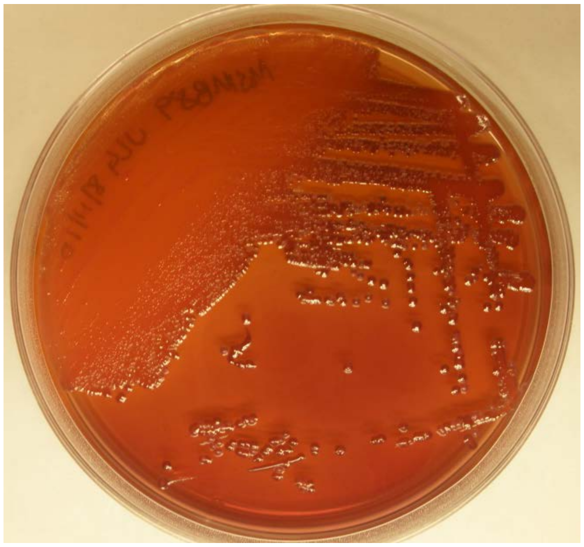

B. ubonensis MSMB89

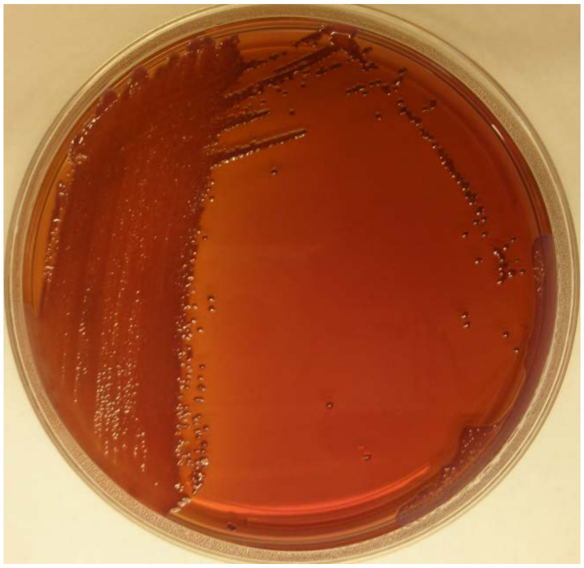

B. ubonensis MSMB65

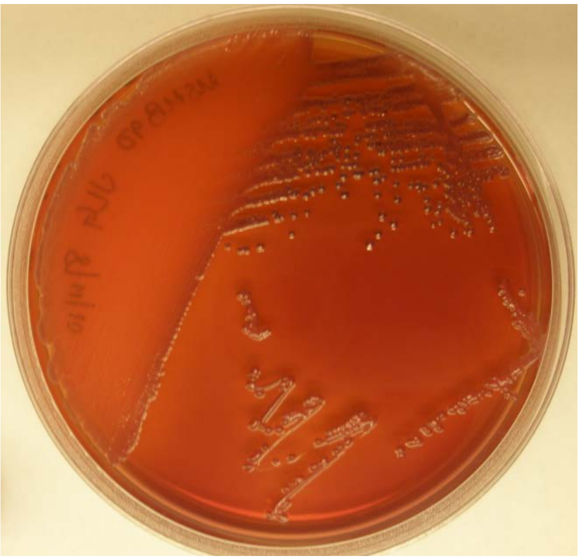

B. ubonensis MSMB90

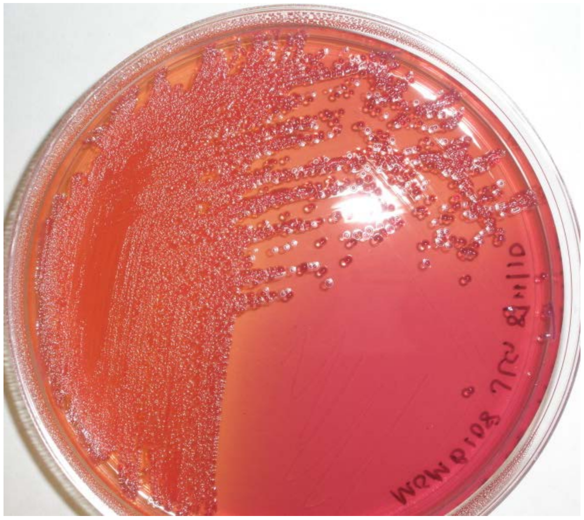

B. ubonensis MSMB108

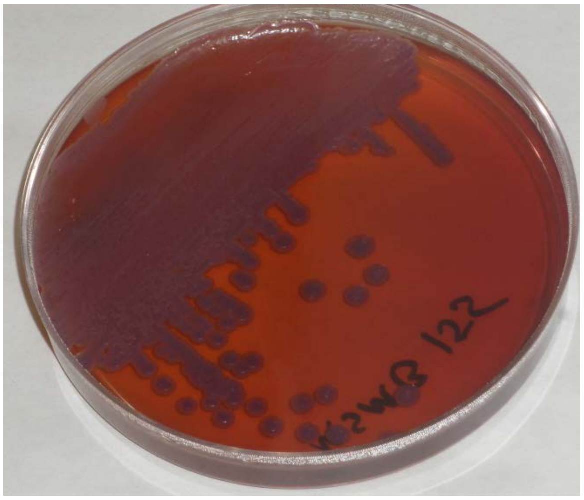

B. ubonensis MSMB155

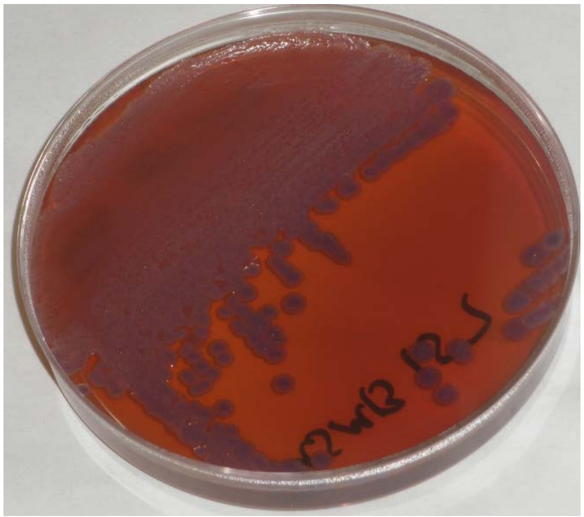

B. ubonensis MSMB157

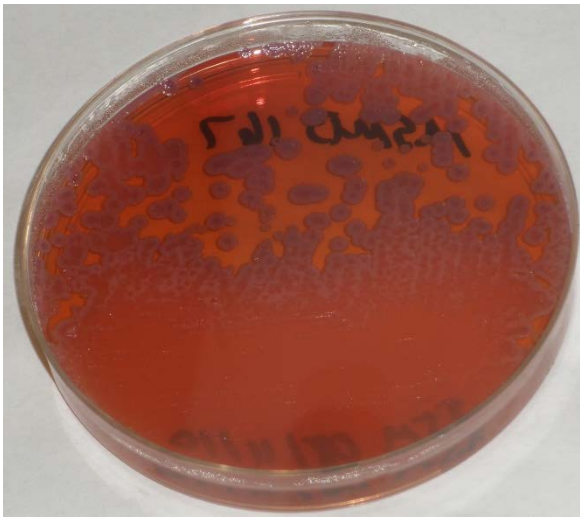

B. ubonensis MSMB167

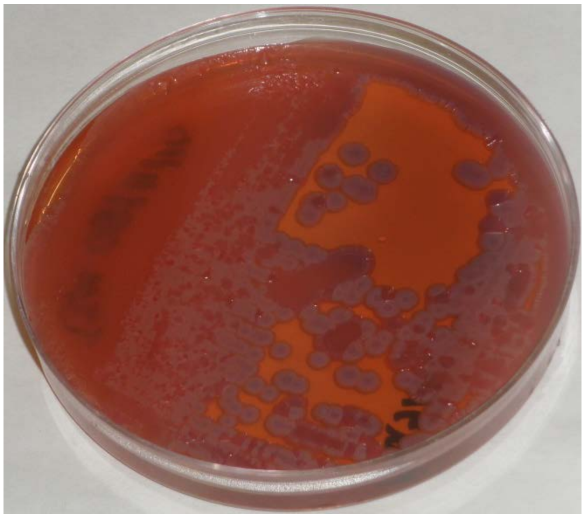

B. ubonensis MSMB170

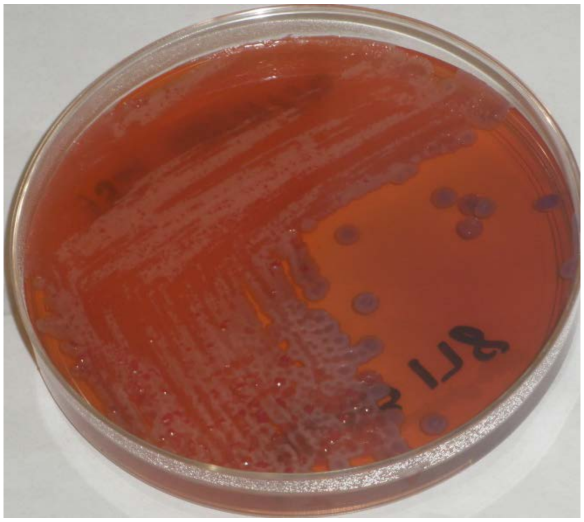

B. ubonensis MSMB178

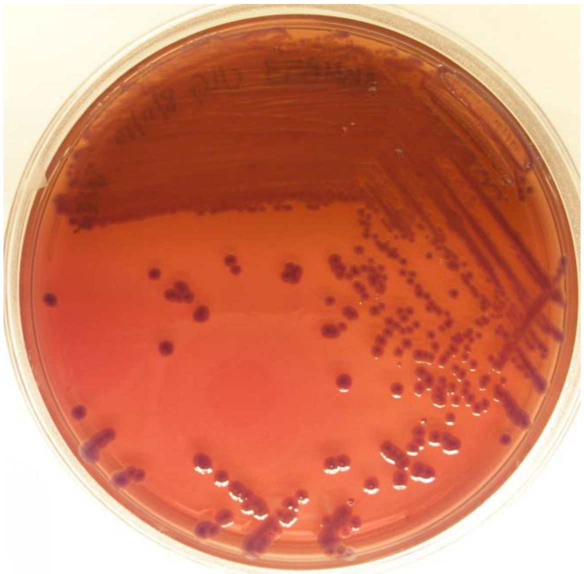

Delftia spp. MSMB53

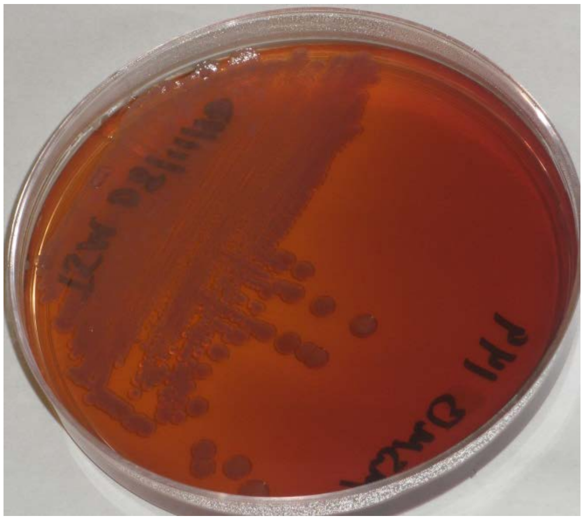

Pandoraea spp. MSMB149
